# Clinical impact of hepatitis delta virus coinfection on liver fibrosis in hepatitis B patients: a population-based study

**DOI:** 10.1186/s12985-025-02908-2

**Published:** 2025-08-09

**Authors:** Sisi Yang, Qiaofeng Ye, Yida Yang, Zhenxuan Ma

**Affiliations:** 1 Department of Infectious Diseases, The First Affiliated Hospital, School of Medicine, Zhejiang University, Hangzhou, China; 2 Department of Biobehavioral Health, Pennsylvania State University, University Park, PA USA; 3 Department of Epidemiology and Biostatistics, Indiana University Bloomington, Bloomington, IN USA

**Keywords:** Hepatitis b virus, Hepatitis d virus, Liver fibrosis, National health and nutrition examination survey, Propensity score matching, APRI, FIB-4, Viral hepatitis

## Abstract

**Background:**

Evaluation of liver fibrosis appears to be easily overlooked in the clinic for the chronic Hepatitis D. Herein, we determine the Clinical Impact of liver fibrosis among HBsAg-positive/Anti-HDV positive US general adults.

**Methods:**

Data were obtained from the National Health and Nutrition Examination Survey (NHANES) 1999–2020. Liver fibrosis was assessed by the Fibrosis-4 (FIB-4) and aspartate aminotransferase to platelet ratio index (APRI) score. To minimize confounding, propensity score matching (PSM) was applied to compare HBV/HDV-coinfected and HBV-monoinfected individuals.

**Results:**

Out of 107,622 NHANES adults, 54,550 were tested for HBsAg and Anti-HDV, of whom 214 were identified as HBsAg-positive only, 50 were identified as HBsAg-positive/Anti-HDV-positive with available data for FIB-4/APRI. Mean APRI scores were 0.26 for the Non-Viral Hepatitis (NVH) group, 0.37 for the HBV group, and 0.42 for the HBV + HDV group. Corresponding mean FIB-4 scores were 1.07, 1.34, and 1.58, respectively. After PSM, HDV-infected individuals exhibited significantly higher fibrosis scores compared to HBV-positive individuals.

**Conclusion:**

Hepatitis D is more severe than hepatitis B, with a higher propensity to progress to liver fibrosis. These findings highlight the importance of routine fibrosis screening in HBsAg-positive/anti-HDV-positive individuals to prevent advanced liver disease.

**Supplementary Information:**

The online version contains supplementary material available at 10.1186/s12985-025-02908-2.

## Introduction

Hepatitis D virus (HDV) is a defective human RNA virus whose replication and transmission depend on the envelope protein provided by hepatitis B surface antigen (HBsAg) [1]. HDV infection manifests in two forms: coinfection and superinfection [2]. Coinfection occurs when HDV and HBV are acquired simultaneously, typically resulting in a self-limiting clinical course accompanied by acute hepatitis. Superinfection, on the other hand, involves HDV infection in individuals who are already HBV carriers, often leading to chronic hepatitis and accounting for approximately 90% of chronic hepatitis D cases [3]. With the implementation of hepatitis B vaccination programs, the global prevalence of HDV infection has significantly declined, particularly in middle- and high-income countries [4]. In the United States, the prevalence of HBV carriers was only 0.36% between 2011 and 2016. However, 42% of these carriers had HDV antibodies, with the majority being intravenous drug users or individuals from regions with high HDV endemicity [5].

Research indicates that chronic hepatitis D is more severe than hepatitis B, with a higher propensity to progress to cirrhosis, hepatocellular carcinoma, and end-stage liver disease [6]. An early study in Italy involving 284 patients with chronic hepatitis D revealed that 93% had active hepatitis or cirrhosis, while only 7% had milder disease [7]. A recent study from Spain showed that among anti-HDV-positive patients, most were young Caucasian males; 60% had advanced fibrosis, and 34% had liver cirrhosis [8]. 

From a public health perspective, determining the burden of liver fibrosis due to hepatitis D is crucial for guiding healthcare resource planning. However, reliable and generalizable data on liver fibrosis among HBsAg positive/anti-HDV positive adults in the United States remain unclear. Utilizing population-based data that can be generalized to the entire U.S. household population, we conducted a preliminary study to investigate the prevalence and predictors of liver fibrosis among HBsAg positive/anti-HDV positive U.S. residents.

## Methods

### Study population

This study utilized public data from the National Health and Nutrition Examination Survey (NHANES), conducted by the National Center for Health Statistics (NCHS), covering the period from 1999 to March 2020 [9]. NHANES employs a stratified, multistage probability sampling design to ensure national representation of the U.S. population. Each biennial survey cycle includes standardized interviews, physical examinations at mobile examination centers, and laboratory tests. Written informed consent was obtained from all participants.

We included individuals aged 18 years or older with complete data for ALT, AST, platelet count, HBV and HDV test results, required for APRI and FIB-4 calculations. Participants with ALT or AST levels exceeding ten times the upper limit of normal and HCV antibody positive were excluded to eliminate cases of potential acute hepatitis or severe hepatitis, requiring liver protection therapy. After applying inclusion and exclusion criteria, a total of 54,550 participants were analyzed. A flowchart of the participant selection process is provided in Supplementary Fig. 1.

### Variable definitions

Key variables in this study were defined based on methodologies established in prior NHANES research [5, 10]. Marital status was categorized as either married/living with a partner or not, while education level was divided into three groups: less than high school, completed high school, and more than high school. Race and ethnicity were classified into five groups: Mexican American, other Hispanic, non-Hispanic White, non-Hispanic Black, and other. Economic status was determined by the Poverty Income Ratio (PIR), with categories for low (< 1), medium [1–3], and high (> 3) income levels. Smoking status was identified as never, former, or current smokers, and alcohol and drug use were dichotomized based on survey responses. HBV vaccination history was recorded as having received 3 doses, 1–2 doses, or no doses.

### Definitions of hepatitis B and D infections and liver fibrosis

Hepatitis B infection was defined by a positive HBsAg result, and HDV infection was defined by a positive HDV antibody result. Based on infection status, participants were grouped into three categories: NVH (individuals without HBV/HDV/HCV infection), HBV (HBsAg-positive only) and HBV + HDV (HBsAg positive and HDV antibody positive). Given that transient elastography results were only available from the cycle after 2017, liver fibrosis status was assessed with APRI [11] and FIB-4 [12] scores calculated as follows:

$$\eqalign{&{\rm APRI (11)}\cr & =\:\frac{\text{A}\text{S}\text{T}(\text{I}\text{U}/\text{L})\times\:100}{\text{U}\text{p}\text{p}\text{e}\text{r}\:\text{l}\text{i}\text{m}\text{i}\text{t}\:\text{o}\text{f}\:\text{N}\text{o}\text{r}\text{m}\text{a}\text{l}\:\text{f}\text{o}\text{r}\:\text{A}\text{S}\text{T}\left(\text{U}\text{L}\text{N}\right)\left(\text{I}\text{U}/\text{L}\right)\text{*}\text{P}\text{l}\text{a}\text{t}\text{e}\text{l}\text{e}\text{t}\:\text{C}\text{o}\text{u}\text{n}\text{t}\left({10}^{9}/\text{L}\right)}}$$

$${\rm FIB-4(12)} =\:\frac{\text{A}\text{g}\text{e}\:\left(\text{y}\text{e}\text{a}\text{r}\text{s}\right)\times\:\text{A}\text{S}\text{T}(\text{I}\text{U}/\text{L})}{\text{P}\text{l}\text{a}\text{t}\text{e}\text{l}\text{e}\text{t}\:\text{C}\text{o}\text{u}\text{n}\text{t}({10}^{9}/\text{L})\times\:\sqrt{\text{A}\text{L}\text{T}(\text{I}\text{U}/\text{L})}}\text{}$$

### Statistical analysis

To account for the extended study period, NHANES weights were recalculated for combined survey cycles, and subsequent analyses used weighted data. The survey package in R was utilized to apply weights appropriately.

Collinearity diagnostics were performed by calculating the Variance Inflation Factor (VIF) for each predictor [13], and no significant collinearity was detected (all VIFs < 5). Linear regression models were used to analyze associations between infection groups (NVH, HBV, HBV + HDV) and fibrosis scores. Regression coefficients (β) indicated the marginal effect of HBV or HBV + HDV infection on APRI and FIB-4 scores, with the NVH group as the reference. A P-trend test assessed the sequential increase in fibrosis scores from NVH to HBV to HBV + HDV groups. Estimated marginal means (emmean) were calculated to assess the significance of differences in APRI and FIB-4 scores between the HBV + HDV and HBV groups.

A multi-model strategy was employed to enhance the robustness of the analysis. Model I was an unadjusted baseline model. Model II included adjustments for demographic factors: age, gender, marital status, education level, birthplace and race/ethnicity. Model III further accounted for lifestyle factors, including smoking, alcohol use, PIR, BMI and drug use. Finally, Model IV incorporated additional adjustments for medical conditions: hypertension, diabetes, HBV vaccine history, kidney disease, heart failure, coronary heart disease, stroke and thyroid disease.

To refine the comparison between HBV and HBV + HDV groups, we performed 1:3 optimal propensity score matching without replacement using the MatchIt package in R [14, 15]. The propensity scores were estimated via logistic regression with the following covariates: Demographics: Age, gender, race, birthplace, marital status; Socioeconomic factors: Education level, PIR. Clinical/lifestyle variables: BMI, hypertension, diabetes, HBV vaccine history, alcohol use, smoking, kidney disease, heart failure, coronary heart disease, stroke and thyroid disease.

Covariate balance was evaluated using standardized mean differences (SMD), with a threshold of |SMD| < 0.1 indicating adequate balance. Balance diagnostics was conducted by visualization in Supplementary Fig. 2. Variables with residual imbalances (SMD ≥ 0.1) were included as covariates in the final post-matching regression model to further mitigate confounding.

Statistical significance was set at *P* < 0.05, and analyses were performed using R version 4.4.1.

## Results

### Participant characteristics

**Fig. 1:**
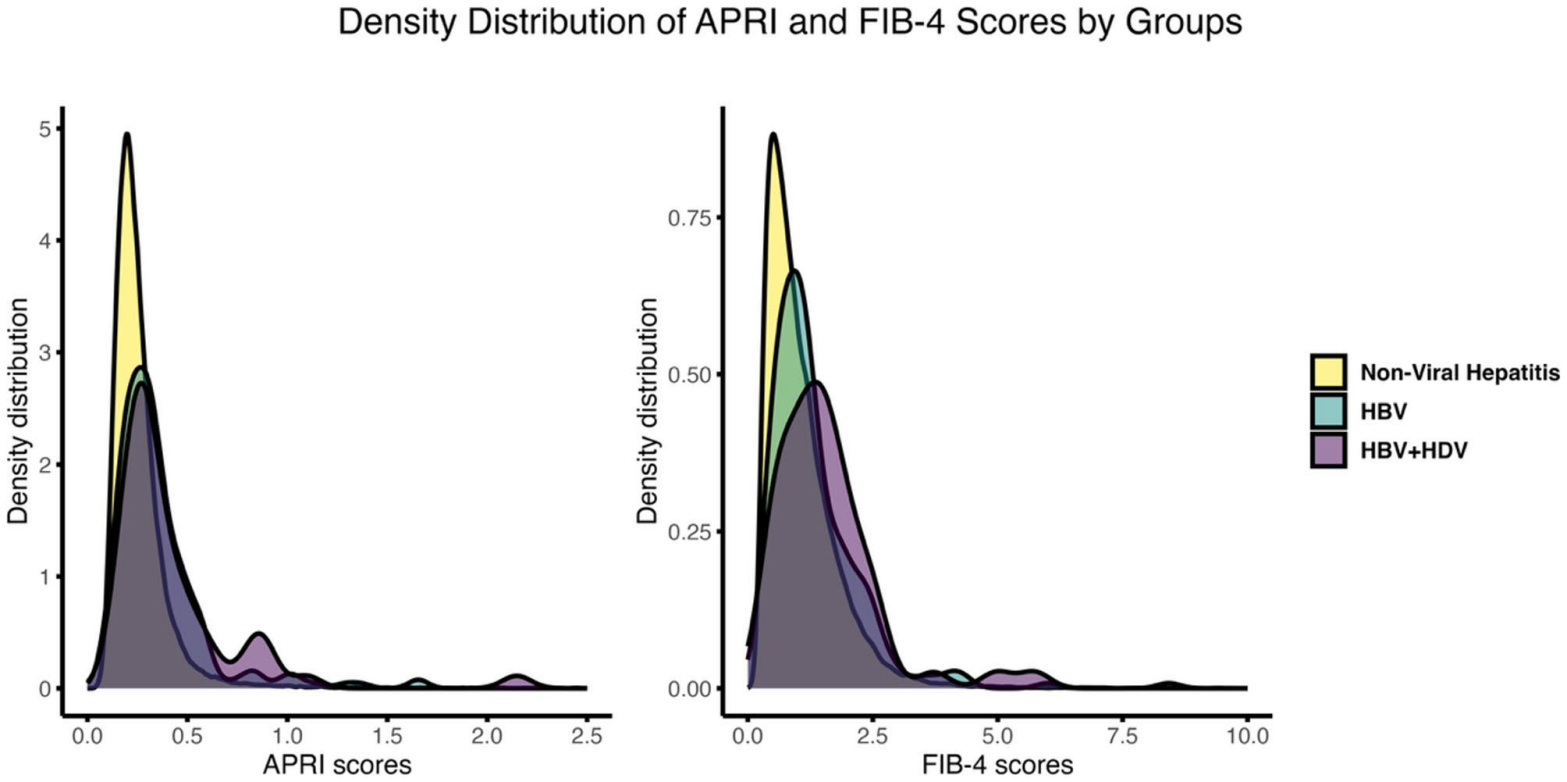
Density distribution of APRI and FIB-4 scores by groups. The density plots compare APRI and FIB-4 scores across Healthy, HBV, and HBV + HDV groups. APRI is a non-invasive marker based on AST and platelet count, while FIB-4 incorporates age, AST, ALT, and platelet count. The X-axis represents the scores, and the Y-axis shows the density distribution. Groups are color-coded, with sample sizes noted in the legend

Of the 54,550 participants analyzed, 214 were HBsAg-positive only, and 50 were HDV-antibody positive, all of whom were also HBsAg-positive. Among HDV-positive participants, 46.0% were male, and 78.0% were born outside the U.S. Mean APRI scores were 0.26 for the NVH group, 0.37 for the HBV group, and 0.42 for the HBV + HDV group. Corresponding mean FIB-4 scores were 1.07, 1.34, and 1.58, respectively. Figure 1 shows density plot of these indices. Detailed participant characteristics are presented in Table 1.

**Table 1:** Baseline characteristics of participants by group

	Non-Viral Hepatitis	HBV	HBV + HDV	*P* value
	*N* = 54,286	*N* = 214	*N* = 50	
Age, years	47.5 (19.2)	48.4 (16.2)	51.0 (16.6)	0.353
Male, n%	26,160 (48.2%)	131 (61.2%)	23 (46.0%)	0.001
Race, n%				< 0.001
Non-Hispanic White	23,432 (43.2%)	24 (11.2%)	5 (10.0%)	
Mexican American	9936 (18.3%)	7 (3.27%)	2 (4.00%)	
Other Hispanic	4566 (8.41%)	9 (4.21%)	1 (2.00%)	
Non-Hispanic Black	11,383 (21.0%)	76 (35.5%)	10 (20.0%)	
Other Race	4969 (9.15%)	98 (45.8%)	32 (64.0%)	
Education level, n%				0.543
< High school	13,527 (25.7%)	62 (29.2%)	14 (28.6%)	
Completed High school	12,770 (24.3%)	48 (22.6%)	8 (16.3%)	
>High school	26,331 (50.0%)	102 (48.1%)	27 (55.1%)	
Married, n%	30,527 (58.6%)	122 (57.8%)	29 (60.4%)	0.943
Birthplace				< 0.001
Not US, n%	14,342 (26.4%)	134 (62.6%)	39 (78.0%)	
PIR				0.209
High income	18,114 (36.7%)	53 (28.8%)	13 (32.5%)	
Medium income	20,527 (41.6%)	83 (45.1%)	19 (47.5%)	
Low income	10,707 (21.7%)	48 (26.1%)	8 (20.0%)	
BMI, kg/m^2^	28.8 (6.87)	26.8 (6.37)	26.0 (5.58)	< 0.001
Alcohol Yes, n%	13,954 (29.5%)	57 (34.5%)	6 (16.7%)	0.087
Smoking				0.341
Never	28,619 (55.7%)	124 (59.3%)	31 (62.0%)	
Former	12,552 (24.4%)	41 (19.6%)	8 (16.0%)	
Current	10,239 (19.9%)	44 (21.1%)	11 (22.0%)	
Drug Yes, n%	11,991 (44.6%)	38 (36.5%)	9 (40.9%)	0.245
HBV Vaccine				0.992
0 dose	38,617 (71.1%)	153 (71.5%)	35 (70.0%)	
3 doses	14,123 (26.0%)	55 (25.7%)	13 (26.0%)	
1–2 doses	1543 (2.84%)	6 (2.80%)	2 (4.00%)	
Hypertension Yes, n%	17,683 (32.7%)	53 (24.8%)	15 (30.0%)	0.045
Diabetes Yes, n%	7649 (14.1%)	40 (18.8%)	5 (10.0%)	0.104
HIV positive, n%	136 (0.48%)	1 (0.89%)	1 (3.57%)	0.072
FIB-4	1.07 (0.82)	1.34 (0.97)	1.58 (1.05)	< 0.001
APRI	0.26 (0.21)	0.37 (0.32)	0.42 (0.33)	< 0.001

Data are presented as mean (standard deviation) for continuous variables or unweighted counts (weighted percentages) for categorical variables. P value shows the differences between groups. One-way ANOVA, Chi-squared Test and Fisher’s Exact Test are used. Missing data were excluded from statistical analysis

FIB-4, fibrosis-4 index; APRI, aspartate aminotransferase to platelet ratio index; BMI, body mass index; PIR, poverty income ratio

### Associations between HDV infection and liver fibrosis

Multivariate regression analyses (Table 2) demonstrated that HDV infection was associated with significantly higher fibrosis scores compared to HBV and NVH groups:

**Table 2:** Multivariate linear regression analysis between APRI/FIB-4 and variables

Variables	APRI				FIB-4			
	Estimate	Std. Error	*P* value		Estimate	Std. Error	*P* value	
Virus Hepatitis								
HBV + HDV	0.238	0.101	0.020	*	0.362	0.111	0.001	**
HBV	0.082	0.023	< 0.001	***	0.158	0.062	0.011	*
Non-Viral Hepatitis	Ref				Ref			
Age	0.001	0.00007	< 0.001	***	0.028	0.0003	< 0.001	***
Gender								
Male	0.066	0.003	< 0.001	***	0.107	0.008	< 0.001	***
Female	Ref				Ref			
BMI	-0.0004	0.0002	0.042	*	-0.009	0.0005	< 0.001	***
Race								
Mexican American	0.017	0.004	< 0.001	***	-0.001	0.009	0.899	
Non-Hispanic Black	0.001	0.003	0.738		0.056	0.009	< 0.001	***
Other Hispanic	-0.002	0.004	0.571		-0.028	0.012	0.026	*
Other Race	-0.003	0.004	0.567		-0.015	0.013	0.231	
Non-Hispanic White	Ref				Ref			
Alcohol								
Yes	0.020	0.003	< 0.001	***	0.033	0.008	< 0.001	***
No	Ref				Ref			
Smoking								
Former	-0.0006	0.003	0.839		-0.007	0.009	0.430	
Current	-0.001	0.004	0.001	**	-0.052	0.009	< 0.001	***
Never	Ref				Ref			

The analysis was performed using weighted multivariate linear regression to account for the complex NHANES sampling design. APRI and FIB-4 are non-invasive indices used to evaluate liver fibrosis, with higher values indicating greater fibrosis severity. Data are retained as three decimal places, some extremely small data retained one significant digit

*P*-values indicate the level of statistical significance, where *** represents *P* < 0.001, ** represents *P* < 0.01, * represents *P* < 0.05, and. represents *P* < 0.1

APRI: HBV + HDV (β = 0.238; 95% CI: 0.137–0.339) vs. HBV (β = 0.082; 95% CI: 0.059–0.105).

FIB-4: HBV + HDV (β = 0.362; 95% CI: 0.251–0.473) vs. HBV (β = 0.158; 95% CI: 0.096–0.220).

Additionally, increasing age, male sex, Mexican American ethnicity, and alcohol use were associated with higher APRI scores. Increasing age, male sex, alcohol use, and Non-Hispanic Black ethnicity were also associated with higher FIB-4 scores.Conversely, higher BMI and current smoking were associated with lower APRI and FIB-4 scores (all *P* < 0.05). However, their respective effect sizes (as indicated by the β coefficients/estimates in Table 2) were notably smaller compared to the impact of viral hepatitis status.

HDV coinfection was associated with a substantially higher risk of liver fibrosis compared to HBV infection alone or the absence of infection. While HBV infection significantly increased fibrosis indices relative to the NVH reference group, the effect size was notably smaller than that observed in the HBV + HDV group. These findings indicated that HDV coinfection exacerbated liver damage beyond the impact of HBV alone, underscoring its role as a critical contributor to liver disease progression. It also illustrated the greater increase in fibrosis indices in the HBV + HDV group compared to the HBV group, emphasizing the heightened clinical burden posed by HDV coinfection in hepatitis B patients. This highlighted the need for targeted interventions and closer monitoring of liver fibrosis in HDV-infected individuals.

**Fig. 2:**
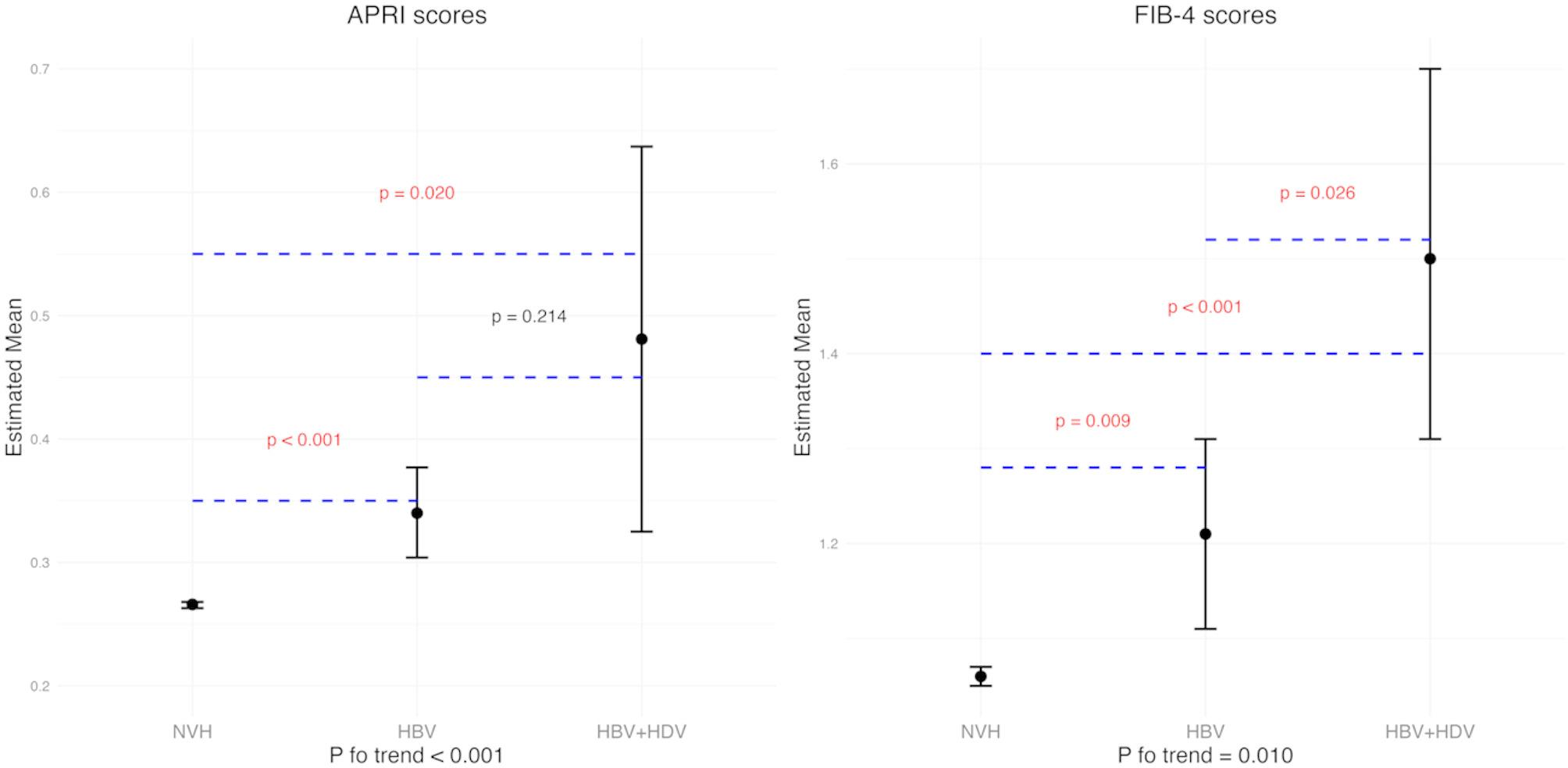
Estimated Means and 95% Confidence Intervals of APRI and FIB-4 Across Different Groups. Estimated means are presented with 95% confidence intervals. P-values indicate pairwise comparisons between groups, and P for trend evaluates the overall trend across the groups

P-trend tests confirmed an incremental increase in fibrosis scores across groups (NVH → HBV → HBV + HDV). Pairwise comparisons using estimated marginal means in Fig. 2 showed significantly higher FIB-4 scores in the HBV + HDV group compared to the HBV group (emmean: 1.50 vs. 1.21, *P* = 0.026). While the HBV + HDV group also exhibited higher estimated marginal mean APRI scores compared to the HBV group, this difference did not reach statistical significance (emmean:0.481 vs. 0.340, *P* = 0.214).

The multi-model analyses showed in Table 3 confirmed that the observed associations remained robust across all four models, even as additional confounding factors were progressively accounted for. Each successive model—from unadjusted to fully adjusted—consistently demonstrated the significant impact of HDV infection on liver fibrosis indices. Furthermore, P-trend tests conducted at each model stage consistently supported the conclusion that fibrosis indices increased sequentially from the NVH group to the HBV group and, finally, to the HBV + HDV group.

**Table 3:** Multivariate association of HBV/HDV infections with APRI and FIB-4 scores

	Model I		Model II		Model III		Model IV	
**APRI**	β (Std. Error)	P	β (Std. Error)	P	β (Std. Error)	P	β (Std. Error)	P
HBV + HDV	0.213(0.079)	0.008	0.217(0.086)	0.013	0.107(0.054)	0.049	0.113(0.055)	0.041
HBV	0.084(0.019)	< 0.001	0.072(0.018)	< 0.001	0.075(0.030)	0.014	0.078(0.031)	0.012
*P trend*	< 0.001		< 0.001		0.008		0.007	
**FIB-4**								
HBV + HDV	0.550(0.170)	0.002	0.456(0.109)	< 0.001	0.258(0.123)	0.039	0.267(0.126)	0.036
HBV	0.174(0.066)	0.009	0.144(0.051)	0.006	0.114(0.050)	0.026	0.120(0.052)	0.022
*P trend*	0.001		< 0.001		0.014		0.012	

Model I: Unadjusted. Model II: Adjusted for demographic factors. Model III: Adjusted for lifestyle factors. Model IV: Adjusted for medical conditions

Reference group: Healthy participants with HBsAg-negative and HDV-negative status

### Propensity score matching for HBV + HDV and HBV groups

To specifically compare the liver fibrosis burden between the HBV and HBV + HDV groups, we performed propensity score matching (PSM) with a 1:3 ratio (HBV + HDV: HBV). This matching approach aimed to balance key baseline characteristics between the two groups, reducing potential confounding effects and enabling a more reliable comparison.

After matching, the HBV + HDV group continued to show higher APRI and FIB-4 scores compared to the HBV group (HBV + HDV vs. HBV):

APRI: Mean (0.425 vs. 0.342), Median (0.309 vs. 0.301), Max (2.148 vs. 1.370).

FIB-4: Mean (1.581 vs. 1.256), Median (1.444 vs. 1.080), Max (5.737 vs. 4.321).

Some variables, such as age, gender, race, birthplace, hypertension, diabetes and heart failure, showed residual imbalances with SMD ≥ 0.1 (Supplementary Fig. 2). To address this, we adjusted for these variables in post-matching regression models. Results indicated that the HBV + HDV group had significantly higher APRI and FIB-4 scores compared to the HBV group (Table 4), confirming that HDV infection imposes a greater burden of liver fibrosis. These findings reinforce the validity of the analysis and underscore the more severe impact of HDV coinfection on liver health.

**Table 4:** Multivariate linear regression analysis of APRI and FIB-4 after propensity score matching

Variables	APRI			FIB-4		
	Estimate	Std. Error	*P* value	Estimate	Std. Error	*P* value
Virus Hepatitis						
HBV + HDV	0.056	0.027	0.040	0.153	0.075	0.041
HBV	Ref			Ref		
Age	0.002	0.001	0.058	0.032	0.003	< 0.001
Gender						
Male	0.090	0.034	0.008	0.164	0.094	0.081
Female	Ref			Ref		
Race						
Mexican American	0.084	0.098	0.391	0.448	0.270	0.099
Non-Hispanic Black	-0.050	0.060	0.411	-0.003	0.166	0.989
Other Hispanic	-0.092	0.146	0.530	-0.032	0.404	0.936
Other Race	0.035	0.060	0.561	0.161	0.167	0.336
Non-Hispanic White	Ref			Ref		
Birthplace						
Not US	0.016	0.046	0.729	0.053	0.126	0.672
US	Ref			Ref		
Education level						
>High school	-0.013	0.041	0.746	-0.149	0.112	0.187
Completed High school	-0.056	0.052	0.291	-0.226	0.145	0.121
< High school	Ref			Ref		
Alcohol						
Yes	0.025	0.038	0.515	0.115	0.106	0.279
No	Ref			Ref		
Kidney disease						
Yes	0.081	0.092	0.383	0.053	0.256	0.837
No	Ref			Ref		
Heart failure						
Yes	-0.104	0.100	0.297	-0.149	0.276	0.590
No	Ref			Ref		

The results indicate that HBV + HDV coinfection is significantly associated with higher APRI and FIB-4 scores compared to HBV alone, suggesting a greater liver fibrosis burden in coinfected patients. Data are retained as three decimal places

## Discussion

Globally, at least 12 million individuals with HBV infection are infected with HDV, corresponding to a prevalence of 4.5% among HBsAg-positive population, with higher prevalence in certain geographic areas and populations, especially in Mongolia, the Republic of Moldova and countries in Western and Middle Africa [16]. Within a large and representative U.S. population from the APCD database (covering ~ 80% of insured Americans), the prevalence of HBV/HDV coinfection was 4.6% [17]. The National Health and Nutrition Examination Survey (NHANES) database provides critical insights into the disease burden of HBV and HDV infections in the general population from 1999 to 2020. HDV infection surveillance primarily relies on anti-HDV detection among HBV carriers; however, anti-HDV positivity only indicates prior HDV exposure or ongoing infection. Definitive confirmation of active HDV replication requires HDV RNA testing in anti-HDV-positive individuals. Since HDV-RNA detection is not yet widely available, subject to the limitations of the available data, an estimated 64.2% anti-HDV-positive individuals in the U.S. had viraemic infection (HDV RNA positive) [16].

This study excluded individuals with ALT or AST > 10$\:\times\:$ULN [18, 19], which were considered to have a predisposition to severe hepatitis or the possibility of acute hepatitis, and require liver protection therapy. In this way, liver fibrosis evaluation through FIB-4 and APRI seemed not suitable and may inaccurate. Individuals with HCV antibody positive were also excluded, as the the major cause of viral hepatitis, HCV infection or previous infection will cause severe fibrosis or cirrhosis [20]. Our research period spans over two decades, due to the limitations of different rounds of NHANES database, it is difficult to identify chronic liver diseases such as MASLD, MetALD, ALD, and autoimmune liver disease, so we selected individuals with non-viral hepatitis, namely individuals without HBV/HDV/HCV infection, as the control group in this study, try to elucidate the impact of HBV infection or HBV/HDV coinfection on liver fibrosis. Consequently, the 50 HBsAg-positive/anti-HDV-positive cases analyzed herein represent HDV infections.

Notably, suboptimal HBV vaccination coverage persists in the U.S., according to our study, over 70% of study participants had not received HBV vaccination, this may attribute to low prevalence of HBV infection (around 0.3%) [21] and insufficient prioritization by public health systems. This has contributed to stable HBV/HDV prevalence rates in recent decades [5, 10].

To mitigate bias, due to the limited HDV infection cohort, we quantified and compared fibrosis scores (FIB-4 and APRI) instead of using the established APRI and FIB-4 thresholds across HDV-infected, HBV-infected only, and NVH groups. Multivariate analyses confirmed significantly higher fibrosis scores in the HDV group. A key strength of our analysis lies in the application of propensity score matching (PSM) to directly compare liver fibrosis indices between HDV and HBV groups while minimizing baseline confounding. Even after matching, HDV-positive individuals exhibited significantly higher fibrosis scores, reinforcing the robustness of our findings. These findings underscore HDV’s distinct and additive role in accelerating liver fibrosis, beyond the effect of HBV alone, consistent with prior evidence that chronic HDV infection accelerates progression to cirrhosis and hepatocellular carcinoma [6, 22], which may be explained by HDV-driven synergistic activation of HBV X (HBx)-mediated TGF-β and c-Jun signaling cascades, promoting hepatic fibrogenesis [23, 24].

These findings supported that routine (every 6 to 12 months) fibrosis screening on liver [25], such as fibroscan or computed tomography scan or Magnetic Resonance Imaging in HBsAg-positive/anti-HDV-positive individuals was supposed to be carried out to early distinguish severe fibrosis or cirrhosis, intervene timely and prevent advanced liver disease.

Importantly, HDV infection is strongly associated with high-risk behaviors (e.g., intravenous drug use, unprotected sex) [4], often co-occurring with HCV, HIV infection and drug abuse-related complications, which further elevates fibrosis risk, NHANES may not fully capture this aspect of the data, from our data only 2 cases were HBsAg-positive/anti-HDV-positive/anti-HCV-positive, 1 case was HBsAg-positive/anti-HDV-positive/anti-HIV positive. To be the low-prevalence country for Hepatitis D, The American Association for the Study of Liver Diseases recommends anti-HDV testing for high-risk HBV carriers in the U.S., While it is reported that HDV prevalence in HBsAg-positive hepatology clinic attendees is reported obviously higher than general people [16], anti-HDV testing was supposed to be carried out among HBsAg-positive hepatology clinic. As a major immigrant-receiving nation, the United States may receive immigrants from HDV endemic regions (e.g., Asia, Africa), It was reported that 31% of US immigrants in 2021 were from Asia according to the data from the Migration Policy Institute [26], from our study, 78.0% of the anti-HDV positive individuals were born outside the U.S., indicating that the majority of anti-HDV positive individuals were imported, so it is also supposed to carry out anti-HDV testing among new immigrants every year with HBsAg-positive. And HDV RNA testing for all anti-HDV positive individuals was supposed to be required. Finally, equitable access to preventive HBV vaccination remains pivotal for HBV/HDV elimination efforts.

However, several limitations should be acknowledged. First, NHANES lacks HDV RNA testing, restricting our ability to distinguish between past HDV exposure and active viral replication. Second, NHANES may underrepresent high-risk populations, such as people who inject drugs and incarcerated individuals, potentially leading to an underestimation of HDV burden. Third, while PSM effectively reduced confounding, residual imbalances remained for certain variables. We employed a doubly robust approach by including these specific variables as covariates in our final post-matching regression models. Although this statistical adjustment is a standard and effective method for controlling for residual confounding, we cannot entirely exclude the possibility that these imbalances or other unmeasured factors may still have subtly influenced our results. Finally, the relatively small number of HDV-positive cases limited statistical power for subgroup analyses. Future studies incorporating clinical cohorts with HDV RNA testing and larger, more diverse populations are warranted to validate and extend our findings.

In summary, our study underscores the substantial burden of liver fibrosis in HBV/HDV-coinfected individuals, providing compelling evidence that HDV coinfection significantly accelerates hepatic fibrosis progression beyond the impact of HBV alone. The findings emphasize the urgent need for enhanced HDV surveillance, routine fibrosis assessment, and targeted interventions to mitigate the long-term hepatic complications associated with HDV infection.

## Supplementary Information

Below is the link to the electronic supplementary material.

Supplementary Material 1

## Data Availability

The data used in this study are publicly available on the NCHS website (https://www.cdc.gov/nchs/nhanes/).

## Abbreviations

NHANES: National Health and Nutrition Examination Survey
NCHS: National Center for Health Statistics
NVH: Non-Viral Hepatitis
HBsAg: Hepatitis B surface antigen
HDV: Hepatitis D virus
HBV: Hepatitis B virus
APRI: Aspartate aminotransferase to platelet ratio index
FIB-4: Fibrosis-4 score
ALT: Alanine aminotransferase
AST: Aspartate aminotransferase
PIR: Poverty Income Ratio
PSM: Propensity Score Matching

## References

[1] Urban S, Neumann-Haefelin C, Lampertico P. Hepatitis D virus in 2021: virology, immunology and new treatment approaches for a difficult-to-treat disease. Gut. 2021;70(9):1782–94. 34103404 10.1136/gutjnl-2020-323888 PMC8355886

[2] Farci P, Niro GA. Clinical features of hepatitis D. Semin Liver Dis. 2012;32(3):228–36. 22932971 10.1055/s-0032-1323628

[3] Kamili S, Drobeniuc J, Mixson-Hayden T, Kodani M. Delta hepatitis: toward improved diagnostics. Hepatology. 2017;66(6):1716–8. 28961326 10.1002/hep.29564

[4] Asselah T, Rizzetto M, Hepatitis D, Virus Infection. N Engl J Med. 2023;389(1):58–70. 37407002 10.1056/NEJMra2212151

[5] Patel EU, Thio CL, Boon D, Thomas DL, Tobian AAR. Prevalence of hepatitis B and hepatitis D virus infections in the united states, 2011–2016. Clin Infect Dis. 2019;69(4):709–12. 30605508 10.1093/cid/ciz001 PMC6669285

[6] Fattovich G, Giustina G, Christensen E, Pantalena M, Zagni I, Realdi G, et al. Influence of hepatitis delta virus infection on morbidity and mortality in compensated cirrhosis type B. The European concerted action on viral hepatitis (Eurohep). Gut. 2000;46(3):420–6. 10673308 10.1136/gut.46.3.420 PMC1727859

[7] Rosina F, Conoscitore P, Cuppone R, Rocca G, Giuliani A, Cozzolongo R, et al. Changing pattern of chronic hepatitis D in Southern Europe. Gastroenterology. 1999;117(1):161–6. 10381923 10.1016/s0016-5085(99)70563-9

[8] Palom A, Rando-Segura A, Vico J, Pacín B, Vargas E, Barreira-Díaz A, et al. Implementation of anti-HDV reflex testing among HBsAg-positive individuals increases testing for hepatitis D. JHEP Rep. 2022;4(10):100547. 36052219 10.1016/j.jhepr.2022.100547 PMC9425021

[9] CDC. National Center for Health Statistics (NCHS). National Health and Nutrition Examination Survey Data. Accessed July 4th, 2025 [Available from: https://wwwn.cdc.gov/nchs/nhanes/default.aspx

[10] Njei B, Do A, Lim JK. Prevalence of hepatitis delta infection in the united states: National health and nutrition examination survey, 1999–2012. Hepatology. 2016;64(2):681–2. 26453027 10.1002/hep.28279 PMC4826621

[11] Wai CT, Greenson JK, Fontana RJ, Kalbfleisch JD, Marrero JA, Conjeevaram HS, et al. A simple noninvasive index can predict both significant fibrosis and cirrhosis in patients with chronic hepatitis C. Hepatology. 2003;38(2):518–26. 12883497 10.1053/jhep.2003.50346

[12] Sterling RK, Lissen E, Clumeck N, Sola R, Correa MC, Montaner J, et al. Development of a simple noninvasive index to predict significant fibrosis in patients with HIV/HCV coinfection. Hepatology. 2006;43(6):1317–25. 16729309 10.1002/hep.21178

[13] Fox JWS, An R. Companion to Applied Regression. Third edition ed. Thousand Oaks CA: Sage; 2019.

[14] Austin PC. An introduction to propensity score methods for reducing the effects of confounding in observational studies. Multivar Behav Res. 2011;46(3):399–424. 10.1080/00273171.2011.568786 PMC3144483 21818162

[15] Imai KKG, Stuart EA. MatchIt: nonparametric preprocessing for parametric causal inference. J Stat Soft. 2011;42(8):1–28.

[16] Stockdale AJ, Kreuels B, Henrion MYR, Giorgi E, Kyomuhangi I, de Martel C, et al. The global prevalence of hepatitis D virus infection: systematic review and meta-analysis. J Hepatol. 2020;73(3):523–32. 32335166 10.1016/j.jhep.2020.04.008 PMC7438974

[17] Gish RG, Jacobson IM, Lim JK, Waters-Banker C, Kaushik A, Kim C, et al. Prevalence and characteristics of hepatitis delta virus infection in patients with hepatitis B in the united states: an analysis of the All-Payer claims database. Hepatology. 2024;79(5):1117–28. 37976395 10.1097/HEP.0000000000000687 PMC11020024

[18] Li F, Qu L, Liu Y, Wu X, Qi X, Wang J, et al. PegIFN alpha-2a reduces relapse in HBeAg-negative patients after nucleo(s)tide analogue cessation: A randomized-controlled trial. J Hepatol. 2025;82(2):211–21. 39094743 10.1016/j.jhep.2024.07.019

[19] Puri P. Acute exacerbation of chronic hepatitis B: the dilemma of differentiation from acute viral hepatitis B. J Clin Exp Hepatol. 2013;3(4):301–12. 25755518 10.1016/j.jceh.2013.08.014 PMC3940633

[20] Younossi ZM, de Avila L, Racila A, Nader F, Paik J, Henry L et al. Prevalence and predictors of cirrhosis and portal hypertension in the united States. Hepatology. 2025. 10.1097/HEP.0000000000001243 39879587

[21] Polaris Observatory C. Global prevalence, cascade of care, and prophylaxis coverage of hepatitis B in 2022: a modelling study. Lancet Gastroenterol Hepatol. 2023;8(10):879–907. 37517414 10.1016/S2468-1253(23)00197-8

[22] Shirvani-Dastgerdi E, Schwartz RE, Ploss A. Hepatocarcinogenesis associated with hepatitis B, delta and C viruses. Curr Opin Virol. 2016;20:1–10. 27504999 10.1016/j.coviro.2016.07.009 PMC5508050

[23] Choi SH, Jeong SH, Hwang SB. Large hepatitis delta antigen modulates transforming growth factor-beta signaling cascades: implication of hepatitis delta virus-induced liver fibrosis. Gastroenterology. 2007;132(1):343–57. 17241884 10.1053/j.gastro.2006.10.038

[24] Roberts AB, Thompson NL, Heine U, Flanders C, Sporn MB. Transforming growth factor-beta: possible roles in carcinogenesis. Br J Cancer. 1988;57(6):594–600. 3044431 10.1038/bjc.1988.135 PMC2246450

[25] European Association for the Study of the L. EASL clinical practice guidelines on hepatitis delta virus. J Hepatol. 2023;79(2):433–60. 37364791 10.1016/j.jhep.2023.05.001

[26] Institute MP. Regions of Birth for Immigrants in the United States, 1960-Present. 2022. Accessed January 14, 2025. [Available from: https://www.migrationpolicy.org/programs/migrants-migration-and-development

