# Supplementary material for: Clinical impact of hepatitis delta virus coinfection on liver fibrosis in hepatitis B patients: a population-based study
Source: Virol J. 2025 Aug 9;22:273. doi: 10.1186/s12985-025-02908-2 (PMC12335798; doi:10.1186/s12985-025-02908-2)
Supplement: Supplementary file 1 — Supplementary Material 1 [file 12985_2025_2908_MOESM1_ESM.docx]

**Supplement table.** Contrast among HBV+HDV/HBV/Non-Viral Hepatitis (NVH) groups

| **Contrast** | **Estimate** | **Std. Error** | **P value** |
| --- | --- | --- | --- |
| **APRI** |  |  |  |
| NVH -HBV | -0.075 | 0.0184 | 0.0002 |
| NVH-(HBV+HDV) | -0.215 | 0.0793 | 0.0200 |
| HBV-(HBV+HDV) | -0.141 | 0.0834 | 0.2138 |
| **FIB-4** |  |  |  |
| NVH -HBV | -0.151 | 0.0504 | 0.0090 |
| NVH-(HBV+HDV) | -0.443 | 0.1010 | 0.0001 |
| HBV-(HBV+HDV) | -0.292 | 0.1120 | 0.0261 |


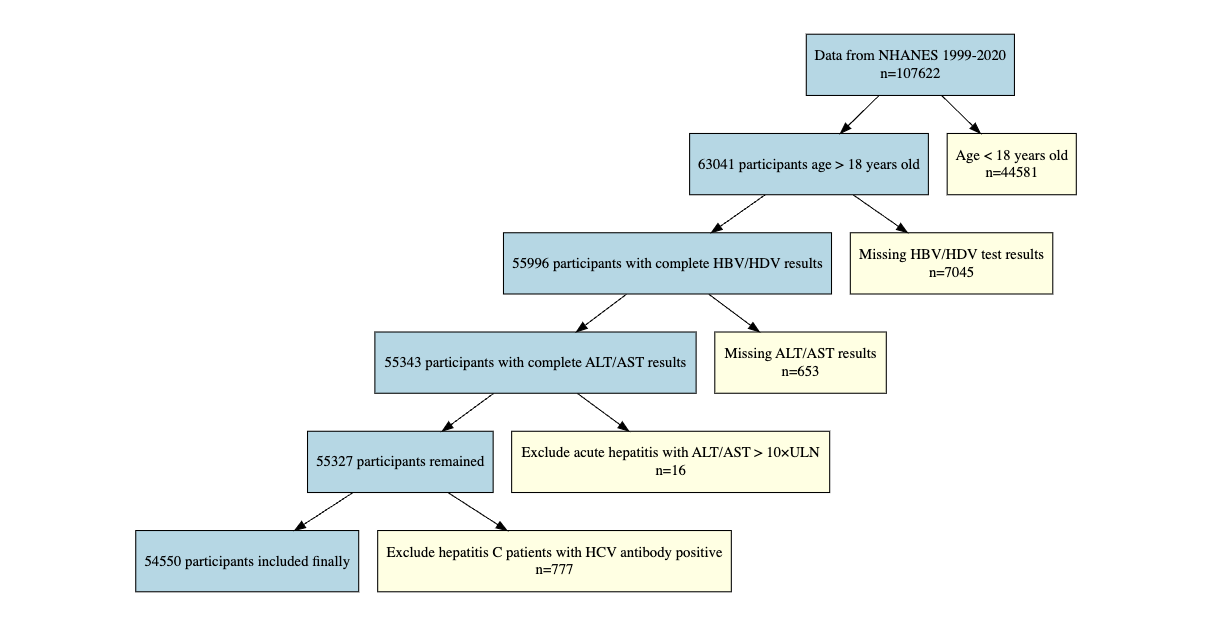


**Supplement Figure 1**. Flow Chart of Participant Inclusion Criteria.


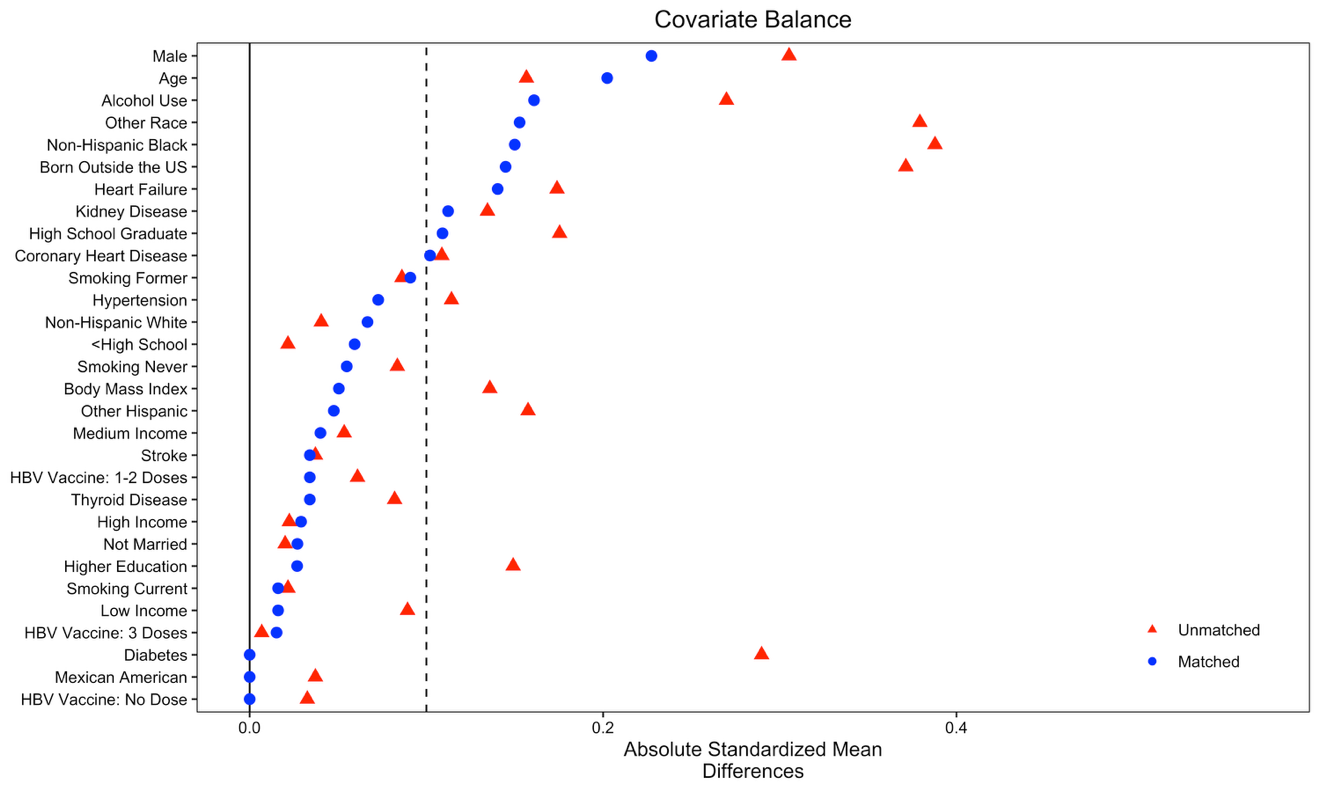


**Supplementary Figure 2**. Covariate balance of propensity scores matching.
